# Dimethyl 3,3′-dithiobispropionimidate-functionalized diatomaceous earth particles for efficient biomolecule separation

**DOI:** 10.1038/s41598-020-72913-8

**Published:** 2020-09-24

**Authors:** Yoon Ok Jang, Geun Su Noh, Huifang Liu, Bonhan Koo, Zhen Qiao, Yong Shin

**Affiliations:** Department of Convergence Medicine, Asan Medical Institute of Convergence Science and Technology (AMIST), University of Ulsan College of Medicine, Biomedical Engineering Research Center, Asan Institute of Life Sciences, Asan Medical Center, 05505 Seoul, Republic of Korea

**Keywords:** Molecular medicine, Diagnostic markers

## Abstract

The early diagnosis and monitoring of cancers are key factors in effective cancer treatment. Particularly, the separation of biomolecules is an essential step for both diagnostic and analytical purposes. However, the current techniques used to isolate biomolecules are intensive, laborious, and require multiple instruments as well as repeated sample preparations to separate each biomolecule. Thus, an efficient separation system that can simultaneously separate biomolecules from scarce samples is highly desirable. Hence, in this study, we developed a biosilica-based syringe filtration system for the efficient separation of biomolecules from cancer samples using amine-modified diatomaceous earth (AD) with dimethyl 3,3′-dithiobispropionimidate (DTBP). The syringe filter can be an efficient and rapid tool for use in various procedures without complex instruments. The DTBP-based AD system was combined with the syringe filter system for nucleic acid and protein separation from various cancer cells. We demonstrated the efficacy of the DTBP-based AD in a single-filter system for the efficient separation of DNA and proteins within 40 min. This DTBP-based AD syringe filter system showed good rapidity, efficiency, and affordability in the separation of biomolecules from single samples for the early diagnosis and clinical analysis of cancers.

## Introduction

Biomaterials for use in the fields of material science, health, and energy have been rapidly developing^1,2^. Moreover, micromaterials and nanomaterials with 3-dimensional (3D) structures have become widely considered to be the most promising candidates for numerous applications in the fields of industrial processing, electronics, diagnosis, and life sciences^3,4^. Nature has mitigated these issues by offering alternative porous-structure biomaterials with multifunctional properties that can be employed using a simple and unique self-assembly process based on accuracy and reproducibility^5,6^. In particular, diatomaceous earth (DE) is a natural, widely available, inexpensive, and multifunctional mineral material that has a 3D porous structure with bio-comfortable silica^7–9^. Moreover, DE is a promising class of porous materials with exceptional surface properties for designing physical and chemical surface modifications and tunable substrates^10–12^. Furthermore, their porous structures have mechanical, photonic, transportable, fabrication, and biocompatibility capabilities, which make this biomaterial suitable for many applications, including biosensing, energy storage, adsorption, purification, photonics, catalysis, bioseparation, and drug delivery^13,14^. Our previous studies have shown the potential of multifunctional materials through the modification of DE composites in biological applications^15–17^.

Bioseparation is significant in the effective and selective isolation and purification of certain biomolecules from complex biological compounds in research and clinical applications, including diagnosis and therapy^18,19^. However, conventional biomolecule separation methods typically cause diagnostic decision errors in which insufficient amounts of acquired biomolecules from rare samples often lead to difficulties in obtaining conclusive results^20–22^. Moreover, each extracted biomolecule divided from heterogeneous samples of evolving diseases, such as cancer, can lead to variable results^21–23^. Accordingly, biomolecule separation methods are important so that downstream analyses can proceed with improved rapidity, efficiency, sensitivity, and specificity across a wide range of biological applications^24,25^. Despite technological advances in the biomolecule separation process, the utility of DE for the simultaneous separation of biomolecules from biological samples has not yet been explored.

Syringe filters are easily manufactured and simple to operate, thus making them applicable to many fields. A large centrifuge is usually essential for analyzing a large volume of samples. However, syringe filters allow large volumes of samples to be analyzed without the use of complex devices, such as large centrifuges or vacuums. Syringe filters have several additional benefits, including the ability to remove outside contamination, organic solvents, and cellular debris from cell lysates^26–28^. Among the various filter membrane materials, polytetrafluorethylene (PTFE) membranes are highly hydrophobic and chemically inert to most organic solvents, alkalis, and acids. PTFE can be used as a filter for aqueous applications^29,30^. Here, we explored efficient processes for biomolecule (protein and nucleic acid) bioseparation from a single specimen using a functionalized DE and syringe filter with a PTFE membrane. To enable the use of DE for this biological application, the DE surface was amine-modified by covalent attachment with 3-aminopropyl(diethoxy)methylsilane (APDMS). Additionally, homobifunctional imidoesters (HIs), including dimethyl adipimidate (DMA), dimethyl pimelimidate (DMP), and dimethyl 3,3′-dithiobispropionimidate (DTBP), were used as non-chaotropic reagents for DNA extraction due to the reversible crosslinking reaction between DNA fragments and the amine groups of functionalized DE with APDMS^31,32^. Thus, we investigated the use of a DTBP-based APDMS-functionalized DE (AD) syringe filter system for the efficient and inexpensive separation of DNA and proteins, thereby streamlining multiplex biomolecule separation. We verified the efficiency of the proposed process of amine-functionalization on the DE surface with APDMS to rapidly and efficiently capture DNA. Moreover, we applied the DTBP-based AD syringe filter system to isolate DNA and proteins from hepatic, prostatic, breast, and colorectal cancer cell lines, as well as a myelogenous leukemia cell line. We validated the utility of the syringe filter system by evaluating the qualities and quantities of the isolated biomolecules compared with the traditional column-based method. Consequently, we demonstrated the application of the DTBP-based AD syringe filter system for isolating biomolecules from a single specimen, thereby providing a convenient approach for the rapid and affordable bioseparation of clinically applicable biomolecules from scarce samples.

## Results and discussion

### Principle of the DTBP-based AD syringe filter system

**Figure 1:**
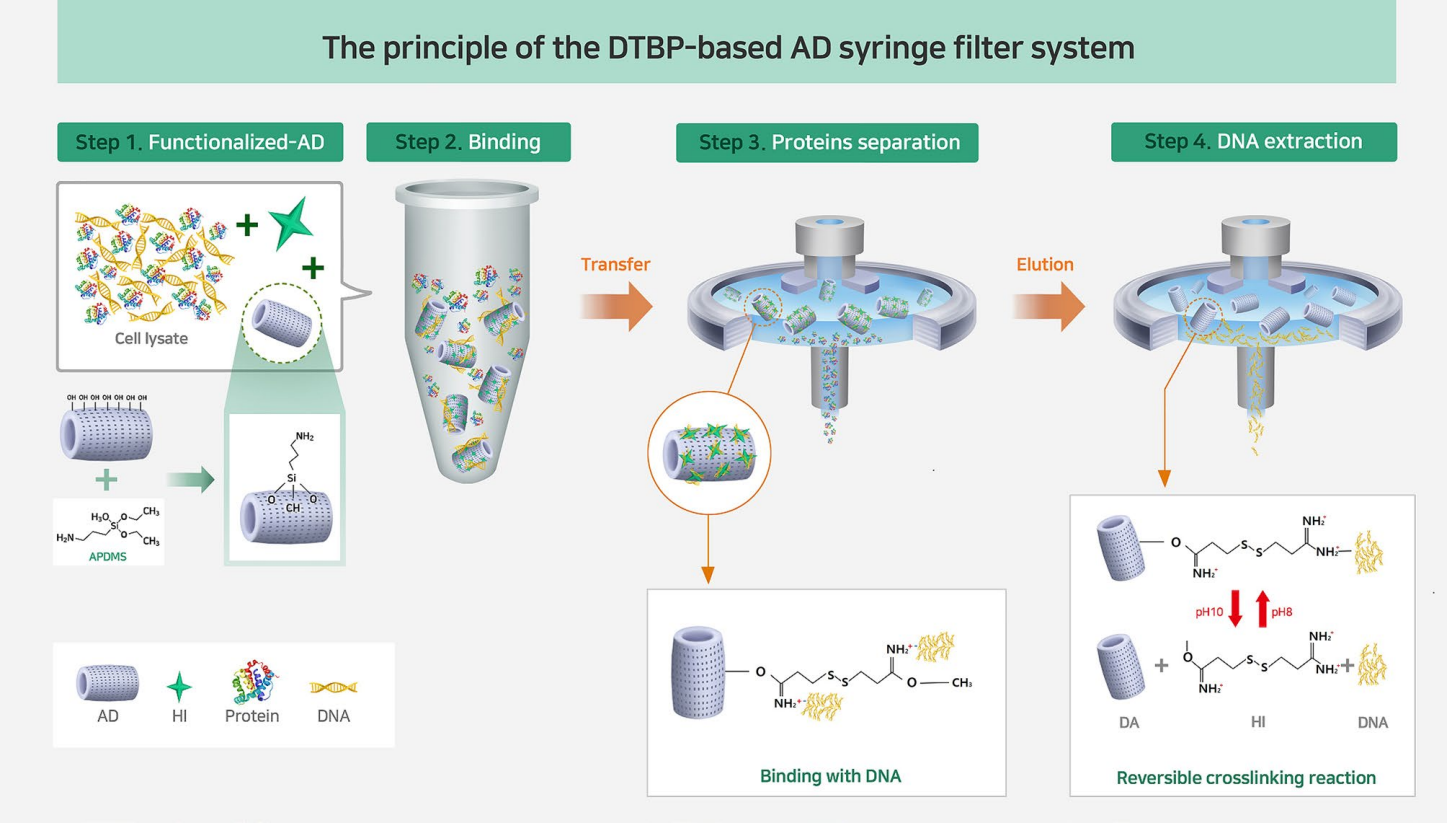
Schematic representation of the principle of the DTBP-based AD in filter system for biomolecule separation. Schematic diagram of the functionalization by APDMS on a DE surface (Step 1). The sample lysate is mixed with DTBP and AD; then, the lysate is incubated to capture DNA via the DTBP on the amine-modified AD surface (Step 2). After this incubation, the protein fractions are directly separated from the single-filter without any additional instruments or detergents (Step 3). Finally, the DNA is isolated from the system with an elution buffer (10 mM sodium bicarbonate, pH 10.6.) (Step 4). (DE: diatomaceous earth, AD: APDMS-functionalized DE).

The principle of the DTBP-based APDMS-functionalized DE (AD) syringe filter system for biomolecule separation is illustrated in Fig. 1. The syringe filter system is composed of AD combined with a DTBP reagent for the separation of both DNA and proteins from a single sample. The DE was modified with APDMS, leading to the formation of an amine-reactive group of the organic molecules (Step 1). After functionalizing AD, DTBP was used as a capture agent for amine group-mediated DNA capture. The DTBP and functionalized AD were mixed with the sample lysate containing RNase for DNA extraction. The DNA captured DTBP on the amine-modified AD surface through both electrostatic interactions and covalent bonds during the 30 min incubation time at RT (Step 2). The proteins were directly separated from the syringe filter after incubation without any binding effect of DTBP (Step 3). The quality and quantity of the isolated proteins were examined using the Bradford assay, Western blotting, and silver staining. Subsequently, the DTBP-based AD in the filter platform was washed thoroughly with PBS using a syringe to remove non-specific molecules and debris. The DNA was extracted with an elution buffer that broke the interaction between the DNA and the DTBP-based AD in the filter platform (Step 4). The quality and quantity of the extracted DNA were examined using a nanodrop spectrophotometer and PCR. Use of the DTBP-based AD syringe filter system allows the separation of DNA and proteins from a single sample without the need for large devices (i.e., large centrifuge), vacuum pumps, or solvents. Moreover, with this system, the DNA and protein templates were successfully isolated within 40 min with simple steps using a common filter and syringe.

### Characterization of the modified-DE with APDMS

**Figure 2:**
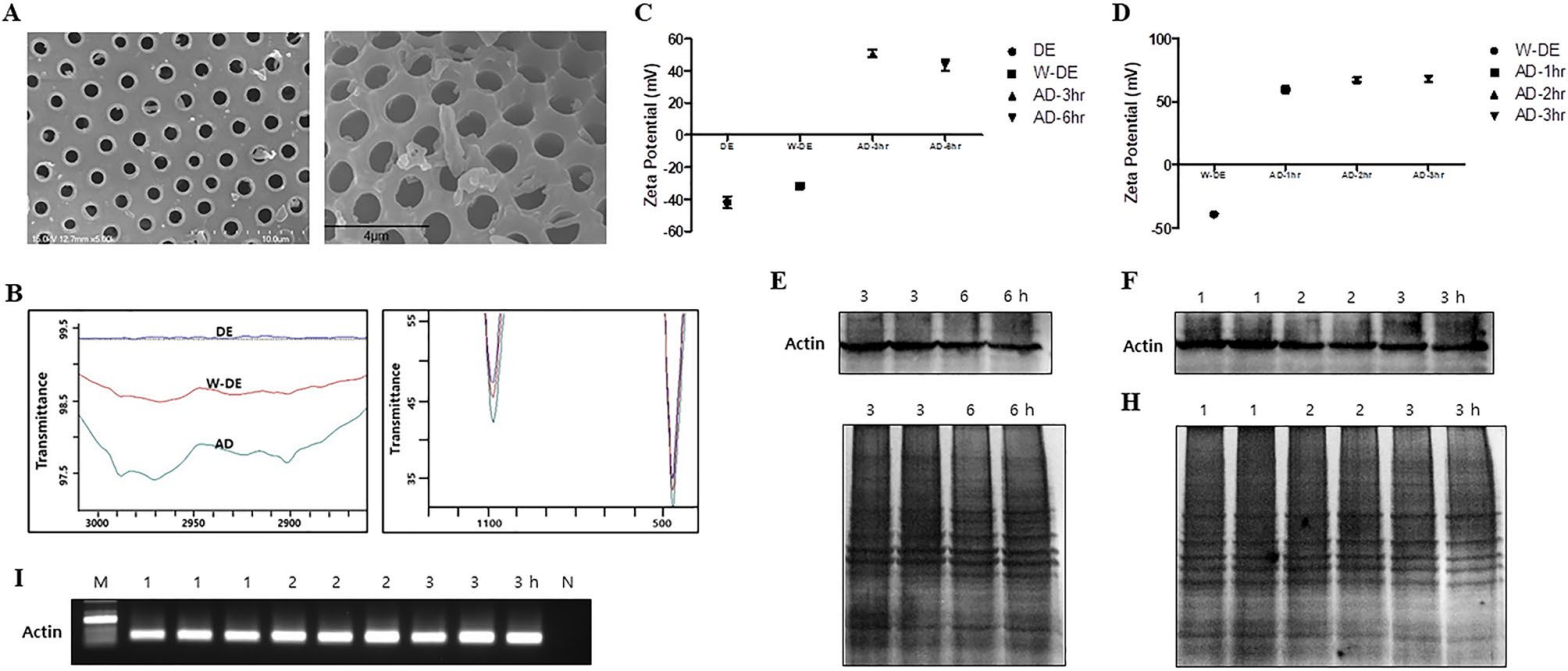
Characterization of APDMS-functionalized DE. (**A**) Scanning electron microscopy (SEM) images of W-DE (left) and AD (right). (**B**) Fourier-transform infrared spectrum analysis of the materials; DE (blue line), W-DE (red line), and AD (green line). (**C**,**D**) Zeta potentials of the prepared materials with different incubation times for DE, W-DE, and AD. Both DNA and proteins from the HCT-116 cancer line (1 × 10^6^ cells) were separated using the DMA-based AD in filter system. (**E**–**H**) Separated proteins were assessed by Western blot for (**E**,**F**) β-actin and by (**G**,**H**) silver staining. (**I**) Extracted DNA was detected by PCR for the *β-actin* gene. (DE: diatomaceous earth, W-DE: washed-DE (unmodified), AD: APDMS-functionalized DE, M: size marker, N: negative control).

The morphologies and pore structures of the washed DE and modified AD were identified in SEM images (Fig. 2A). Additionally, FTIR was used to confirm the functionalized AD. The FTIR spectra of AD are presented in Fig. 2B. The absorption peaks at 1077 and 467 cm^−1^ are ascribed to the asymmetric stretching vibrations of Si–O–Si, and the absorption at 792 cm^−1^ is attributed to Si–O. The band at 618 cm^−1^ has been interpreted as the symmetric stretching vibration of the oxygen atoms located in a ring consisting of three (Si–O) units; its intensity is affected by the OH content in the silica frame. Moreover, the absorption peaks between 2850 and 2980 cm^−1^ are attributed to the symmetric and asymmetric stretching vibrations of the CH_2_ and CH_3_. The absorption bands between 2135 and 2495 cm^−1^ are the characteristic peaks containing C–C and O–H^9,33–36^. The transmittance was higher in the washed DE than the pure DE. Moreover, the transmittance was higher in the modified DE than the washed-DE. Thus, we observed the highest transmittance in the modified AD compared with other materials. These results confirm the modification of amine groups on the surface of pure DE. To optimize the HI-based AD, we evaluated the components and processes in the platform. Typically, the surface modification of AD was performed by the silanization process. First, we tested the modified ADʼs ability at different incubation times (3 and 6 h). Using zeta potential analysis, we observed that the optimal incubation time of 3 h was more efficient than 6 h (Fig. 2C). Moreover, we verified the efficiency of the AD binding time by assessing the ability of the AD at various incubation times (1, 2, and 3 h) using zeta potential analyses. The results did not significantly vary with these different incubation times (Fig. 2D). Furthermore, we performed DMA-based AD at various incubation conditions for DNA and protein bioseparation from 1 × 10^6^ HCT-116 cells. To verify the HI-based AD platform, we first tested this system using DMA out of HIs that we had previously validated^18^. Subsequently, DNA and protein separation using the DMA-based AD filter system was evaluated by Western blot analysis, silver staining, and PCR. Isolated proteins were assessed by Western blot analysis for β-actin (Fig. 2E,F) and silver staining (Fig. 2G,H). Moreover, the extracted DNA was detected by PCR for the *β-actin* gene (Fig. 2I). These results verify that the DMA-based AD filter system for all incubation times could successfully and efficiently separate DNA and proteins from a single sample. Thus, based on the efficiency of the silanization process, the 1 h incubation time for AD modification was considered to be the optimal condition (Fig. 2).

### Optimization of the DTBP-based AD syringe filter system

**Figure 3:**
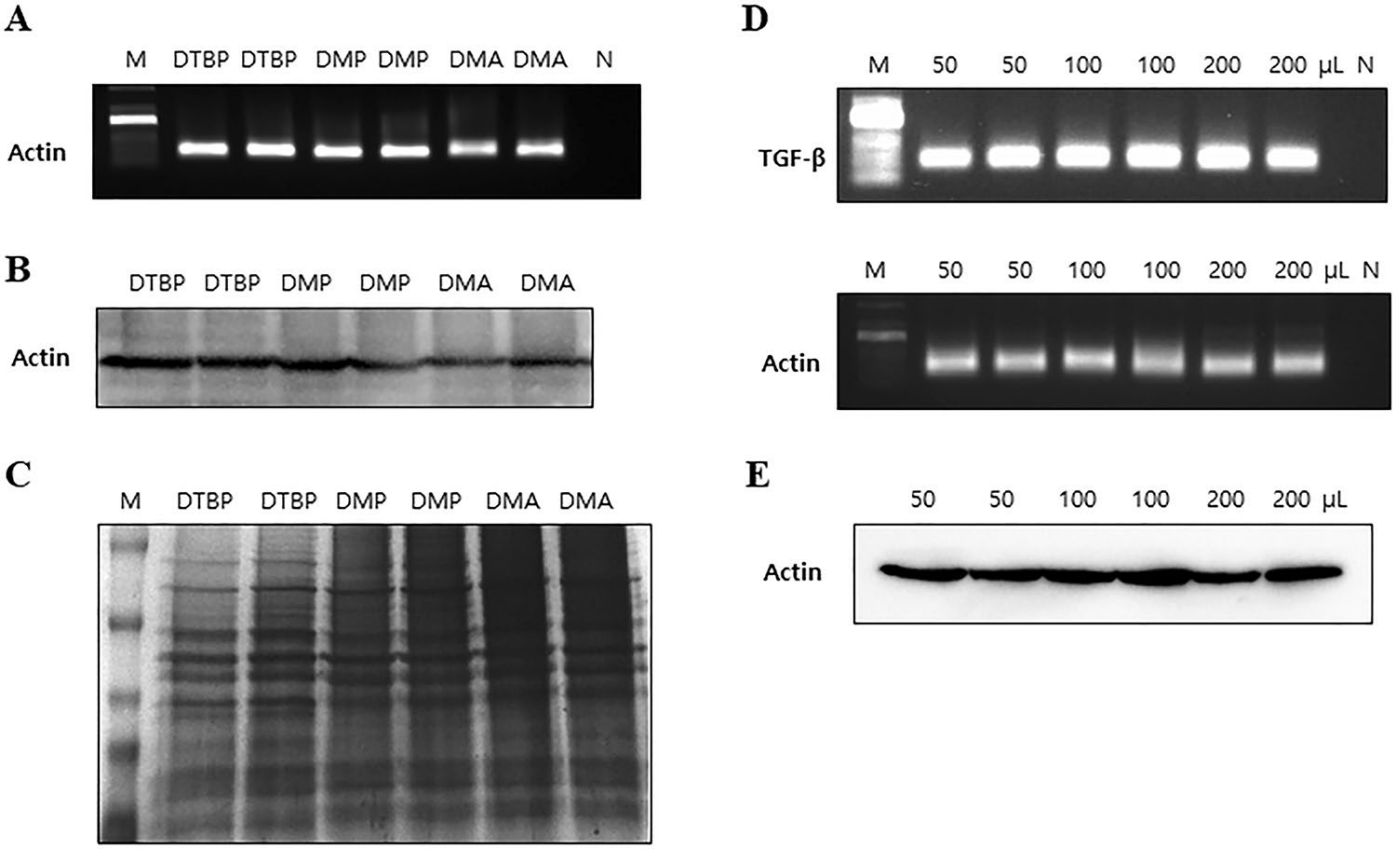
Optimization of the DTBP-based AD syringe filter system. Both DNA and proteins from the HCT-116 cancer line (1 × 10^6^ cells) were isolated using the HI-based AD in filter system. (**A**) PCR analysis of the DNA extracted from HCT-116 cells using this system with different HIs (DTBP, DMP, and DMA). (**B**,**C**) Proteins isolated from the HCT-116 cells using the HI-based AD system were evaluated by (**B**) Western blot and (**C**) silver staining (DTBP, DMP, and DMA). (**D**–**F**) To optimize the DTBP conditions, the DNA and proteins were separated with different volumes of DTBP (50, 100, and 200 μL of 50 mg/mL) were evaluated by (**D**) PCR, (**E**) Western blot, and (**F**) silver staining. (DE: diatomaceous earth, AD: APDMS-functionalized DE, M: size marker, N: negative control).

To evaluate the efficiency of the new HI-based AD platform, we investigated amine-functionalized DE using different HI reagents to directly bind to DNA. To identify the optimal HI reagent, 100 μL of 200 mg/mL HI reagents, including DTBP, DMP, and DMA, were added to the mixture containing 200 μL of 1 × 10^6^ HCT-116 cell lysate and 150 μL of 50 mg/mL AD for DNA and proteins separation. Subsequently, the extracted DNA was evaluated for *β-actin* detection by PCR (Fig. 3A). Separated proteins were assessed for β-actin detection by Western blot (Fig. 3B) and silver staining for whole protein analysis (Fig. 3C). We observed that all HI reagents combined with the AD system could efficiently separate the DNA and proteins (Fig. 3A–C). As a result, DTBP was selected as the optimal HI capture reagent as it had a slightly higher capture efficiency of biomolecules. In addition to optimizing the conditions for DTBP usage, different amounts of DTBP (50, 100, and 200 μL of 50 mg/mL) were examined using the mixture containing 150 μL of 50 mg/mL AD and 200 uL of 1 × 10^6^ HCT-116 cell lysate. The extracted DNA was amplified by PCR for *β-actin* and transforming growth factor-beta (*TGF-β*) (Fig. 3D), and isolated proteins were again used for β-actin detection by Western blot analysis (Fig. 3E). The results were not significantly different in various volumes of DTBP at other concentrations. Based on the ability and efficiency of the DTBP-based AD filter platform, DTBP was chosen as the optimal HI agent at the optimal condition of 50 μL of 50 mg/mL DTBP.

### Characterization of the DTBP-based AD syringe filter system for biomolecule separation

**Figure 4:**
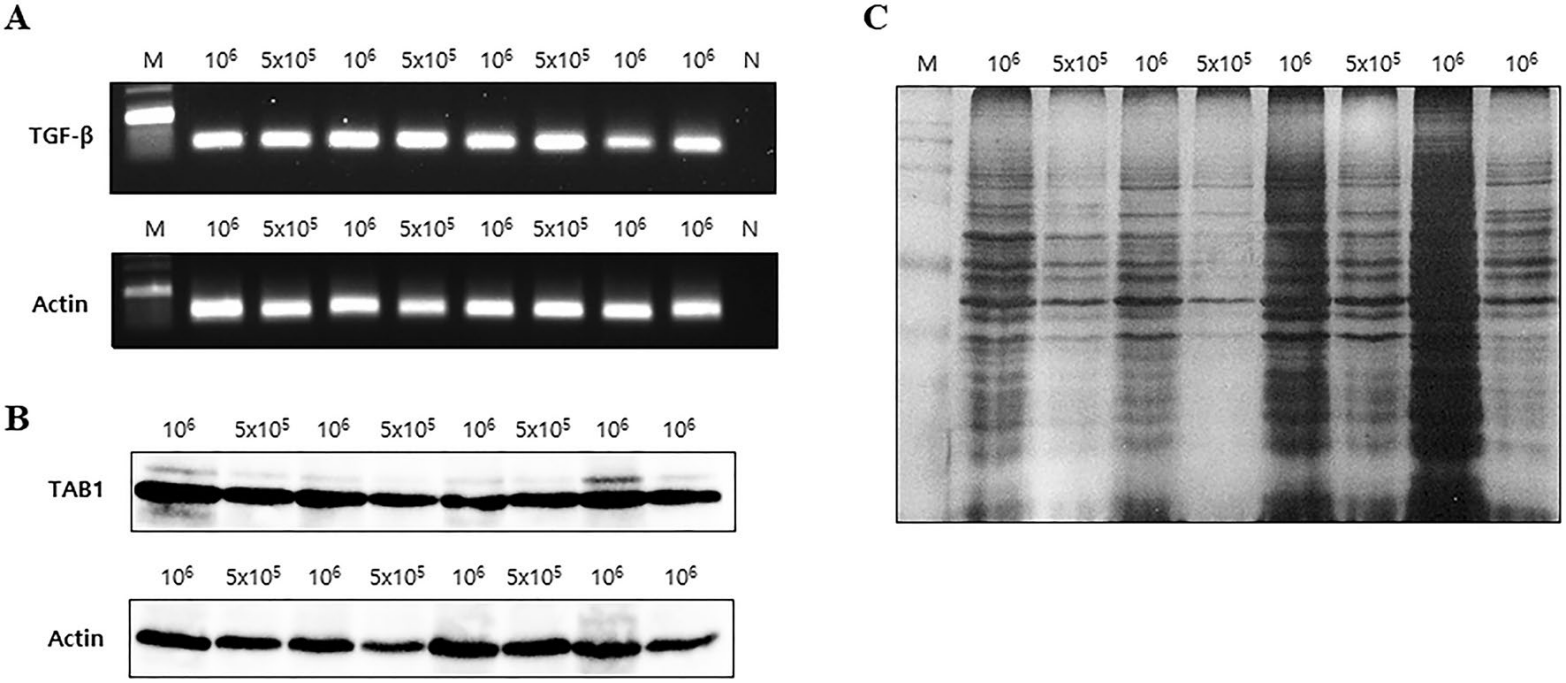
Fundamental characterization of the DTBP-based AD syringe filter system. Both DNA and proteins from various cancer cell lines (5 × 10^5^ and 1 × 10^6^ cells) were isolated using the DTBP-based AD in filter system. (**A**) PCR analysis of the DNA extracted from cancer cells using this system. (**B**,**C**) Proteins separated from the HCT-116 cells using the DTBP-based AD in filter system were evaluated by (**B**) Western blot and (**C**) silver staining. *Lanes 1,2*: HepG cells. *Lanes 3,4*: HCT-116 cells. *Lanes 5,6*: MCF-7 cells. *Lane 7*: LNCap cells. *Lane 8*: K-562 cells. (DE: diatomaceous earth, AD: APDMS-functionalized DE, M: size marker, N: negative control).

Furthermore, to validate the optimal conditions (the mixture containing 150 μL of 50 mg/mL AD, 50 μL of 50 mg/mL DTBP, and 200 μL of 5 × 10^5^ or 1 × 10^6^ cells lysate) for the DTBP-based AD syringe filter system, we assessed DNA and protein bioseparation using various cancer cell lines (Human HepG2, HCT-116, MCF-7, LNCaP, and K-562). The extracted DNA was identified by PCR for *β-actin* and *TGF-β* detection (Fig. 4A), and isolated proteins were identified by Western blot for β-actin and TAB1 (Fig. 4B) selected as testing model proteins in this study and by silver staining (Fig. 4C). TGF-β is a pleiotropic cytokine that mediates a diverse array of physiologic activities in responsive cells and tissues. TGF-β has been implicated in tumorigenesis, and TGF-β-mediated signaling is indeed strongly implicated in cancer regulation^37–39^. Consistent with this notion, increased TGF-β expression in tumor cells correlates with tumor progression in lung carcinoma, colorectal cancer, breast carcinoma, prostate cancer, and gastric carcinoma^37–43^. TAB1 has been known to be critically involved in TGF-β-activated kinase 1 activity and necessary for TGF-β signal transduction^44^. These results indicate that a combination of AD and DTBP can act as a DNA and protein separation method with the assistance of a non-chaotropic agent. In this study, we demonstrated that this DTBP-based AD syringe filter system could isolate DNA as well as proteins from various cancer cell lines. The extracted DNA was identified by PCR for the target genes, and isolated proteins were identified by Western blot analysis for tumor protein markers. These results confirm that the DTBP-based AD syringe filter system can be used to detect target markers of various cancer cells.

### Validation of the DTBP-based AD syringe filter system for DNA and protein separation

**Figure 5:**
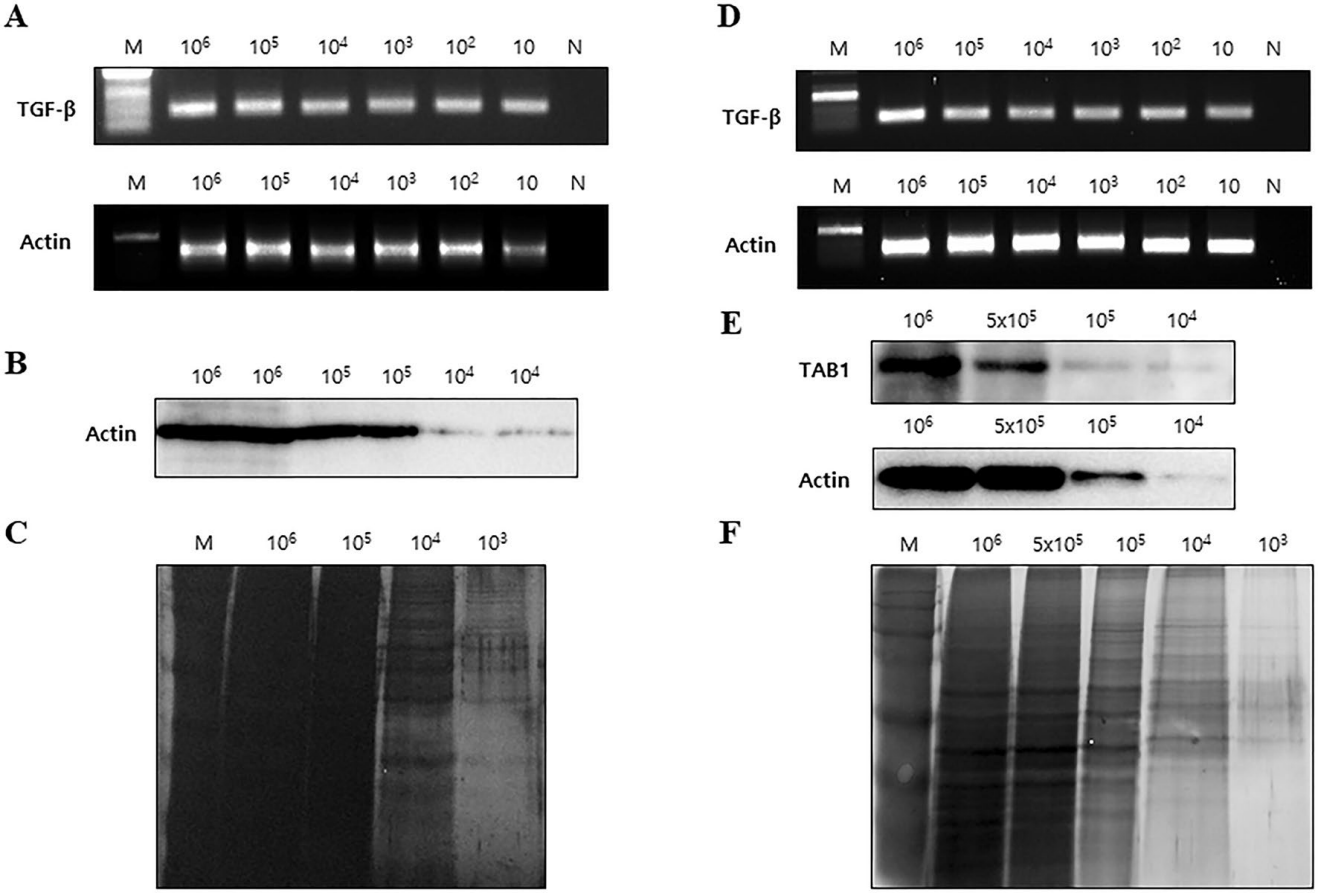
Validation of the DTBP-based AD syringe filter system using LNCap and HepG2 cells. (**A**–**C**) The capacity of the DTBP-based AD in filter system was evaluated with the LNCap cancer cell line, in the range of 10–10^6^ cells. The DNA extracted using the DTBP-based AD in filter system was analyzed by (**A**) PCR for *β-actin* and *TGF-β*. The proteins isolated by the DTBP-based AD in filter system were assessed by (**B**) Western blot for β-actin and (**C**) silver staining. (**D**–**F**) The capacity of the DTBP-based AD in filter system was evaluated with the HepG2 cancer cell line, in the range of 10–10^6^ cells. The DNA extracted using the DTBP-based AD in filter system was analyzed by (**D**) PCR for *β-actin* and *TGF*-*β*. The proteins isolated by the DTBP-based AD in filter system were assessed by (**E**) Western blot for β-actin, TAB1, and (**F**) silver staining. (DE: diatomaceous earth, AD: APDMS-functionalized DE, M: size marker, N: negative control).

To further validate the utility and capacity of the DTBP-based AD syringe filter system, we assessed DNA and protein separation in LNCap and HepG2 cells. First, we validated the DTBP-based AD syringe filter system over a concentration range of 10 to 10^6^ LNCap cells per 200 μL of lysis buffer. The extracted DNA was analyzed by PCR using sequence-specific primers for *β-actin* and *TGF* (Fig. 5A). The evaluation of protein separation was conducted by Western blotting for β-actin (Fig. 5B) and silver staining (Fig. 5C). Moreover, we assessed the DTBP-based AD syringe filter system over a concentration range of 10 to 10^6^ HepG2 cells per 200 μL of lysis buffer. The extracted DNA was analyzed by PCR for *β-actin* and *TGF-β* (Fig. 5D). The isolated proteins were evaluated by Western blotting for β-actin and TAB1 (Fig. 5E) and silver staining (Fig. 5F). Furthermore, to validate the utility of DTBP-based AD through syringe filter systems, we compared them with the column-based method for DNA and protein separation over the concentration range of 10 to 10^6^ LNCap cells. The separated DNA and protein using the column-based system were evaluated by PCR, Western blot analysis, and silver staining (Fig. S1A–C). Similar to the results of the DTBP-based AD syringe filter system, the extracted DNA from the concentration range of 10 to 10^6^ cancer cells using the column-based system was identified by PCR for *β-actin* and *TGF-β* (Fig. 5A,D, and S1A). Moreover, the isolated proteins using the DTBP-based AD syringe filter platform and the column-based method were similar to the Western blot (Fig. 5B,E, and S1B) and silver staining results (Fig. 5C,F, and S1C). The separated protein and DNA from the various cancer cell lines using the DTBP-based AD syringe filter system were evaluated by the NanoDrop UV and the Bradford assay. The concentrations of protein and DNA were approximately 20–30 µg/μL and 20–30 ng/μL, respectively (Table S1). Next, to clear whether the proteins absorb on the modified AD particles, we evaluated the concentration of proteins from both DNA separation (step 4 in Fig. 1) and protein separation (step 3 in Fig. 1) groups using the Bradford protein assay (Fig. S2). The average concentration of proteins in the protein separation group was 26.9 ± 0.51 µg/μL (n = 4) and 0.16 ± 0.01 µg/μL (n = 4) for the protein separation and DNA separation groups, respectively (Fig. S2-right). Hence, the proteins could not absorb on the modified AD particle during the DNA separation process due to the proteins are required relatively longer time to bind with DTBP compared to nucleic acids. Regarding the separation of the biomolecules, the column-based method has complex steps, which require repeated centrifugation steps, followed by the removal of supernatants depending on the type of specimen and additional mechanical treatment. Moreover, the method using radioimmunoprecipitation assay (RIPA) buffer requires more than 30 min for only protein separation. Whereas, with this system, the DNA and protein templates were successfully isolated within 40 min with simple steps using a common filter and syringe. Moreover, the DTBP-based AD syringe filter system has the advantage of low cost and an easy process that uses simpler solvents and steps than the column-based method. Furthermore, upon comparing this system to the column-based method for DNA and protein separation, the results and procedure times were higher (Table S2). These results indicate that using the DTBP-based AD syringe filter system for protein and DNA bioseparation from a single sample can be done rapidly and simply without the need for complex solvents or large instruments.

## Conclusions

We developed the DTBP-functionalized AD through a syringe filter with a PTFE system for DNA and protein separation from a single liquid sample. This system has the advantages of its low cost and an easy process that uses simpler solvents and steps than the column-based method. We validated the utility of the DTBP-functionalized AD syringe filter system approach for the simultaneous bioseparation of proteins and DNA from various cancer cells by detecting target markers associated with tumor development. These results indicate that the DTBP-based AD syringe filter system can conduct on-site analysis without any external devices for various samples through the selection of different syringe sizes and filter types according to the sample volumes. Hence, through this study, we further report the use of an accurate and rapid assay as a diagnostic tool for the analysis of target markers in cancer samples; this tool can provide better information for the early detection and diagnosis of cancer to improve the effectiveness of cancer treatments. Although we have established a proof of concept for the utility of this system for different cancer cell lines, this system will require further verification with clinical sample protocols to improve the versatility and utility of this system in clinical applications. Certainly, methods that simultaneously separate nucleic acids and proteins from the same specimens will be used more frequently as sample preparation tools for various applications in the future. By combining the proposed method with various detection techniques, this DTBP-functionalized AD syringe filter system will also likely improve research quality in the fields of biomedical and biological engineering by reducing the processing times, step complexity, and the need for bulky, complex instruments.

## Materials and methods

### Preparation and functionalization of DE with APDMS

Amine-functionalization of the diatomaceous earth (DE) was performed via a silanization process in which the surface of the DE reacted with the amine group on silane using APDMS (97%, Sigma-Aldrich St. Louis, MO, USA). Biocompatible DE mineral powder (18 μm; Sigma-Aldrich) was washed with distilled water (DW) for 10 min with vigorous stirring. Impurities were removed after brief gravity settling of the mixture. After being washed with 99% ethanol and DW with stirring, clean DE was collected by centrifugation. Two grams of the DE sample was suspended in 25 mL of toluene and stirred for 1 h at 60 °C, and then 0.5 mL of DW was added to this mixture, which was then stirred for 1 h at 65 °C. Subsequently, 1 mL of APDMS was added dropwise to the mixture and stirred for 1 h at 65 °C. The APDMS-DE was collected by centrifugation and washed several times with isopropanol and toluene. Subsequently, the functionalized AD was dried to a desiccator at ambient temperature in a vacuum.

### Characterization of the DE with APDMS

The morphologies of the DE and AD were characterized using field-emission scanning electron microscopy (FE-SEM; JSM-7500F, JEOL, Tokyo, Japan). Fourier-transform infrared (FTIR) spectroscopy (JASCO 6300, JASCO, Easton, MD, USA) analysis was performed on unmodified DE and AD to obtain information on the chemical modifications. The zeta potentials of the material composites were evaluated using dynamic light scattering (DLS, DynaPro NanoStar, Wyatt, GA, USA) to estimate the efficiency of the amino modification.

### Operation of the HI-based AD syringe filter system

The platform could be operated in four steps: (1) HI-based AD platform preparation, (2) sample mixing and binding, (3) protein separation, and (4) DNA extraction (Fig. 1). First, the various incubation times (1, 2, 3, and 6 h) were examined in the AD to set up the optimal conditions for interactions between APDMS and DE. Second, various HIs, such as DMA, DMP, and DTBP, were evaluated in the AD platform for their capability to directly bind to DNA. Third, the optimal concentration of DTBP (50, 100, or 200 μL of 50 mg/mL) was examined via the HI-based AD platform for the amine groups of the AD surface and DNA. Specifically, the platform operation was as follows: (1) after preparing the AD, (2) the AD and DTBP were mixed with the sample lysate. The mixture was incubated for 30 min so that the DNA could bind with DTBP via covalent bonding and electrostatic coupling on the surface. After incubation, the mixture was transferred into the filter via a syringe. (3) The proteins were separated from the mixture in the DTBP-based AD syringe filter platform, and the platform was washed with PBS to remove debris. (4) Finally, DNA was extracted using the elution buffer.

### Optimization of the DTBP-based AD syringe filter system

To optimize the DTBP-based AD system, first, 50 mg of AD was dissolved in 1 mL of DW. Then 50 μL of the 50 mg/mL DTBP solution and 150 μL of 50 mg/mL AD uniformly dispersed suspensions by vortex was added to 200 μL of the sample lysate containing RNase. The solution was mixed and incubated for 30 min at room temperature (RT) on an orbital shaker at 850 rpm to allow the DTBP and DNA complex to bind to the amine-modified surface. After incubation, the sample mixture was transferred into a sterile syringe filter (25 mm diameter with 1 μm pore size; Whatman). The proteins were separated from a single specimen using a syringe on the DTBP-based AD through the syringe filter platform in which proteins were not captured by the DTBP-based AD, while DNA was captured by the DTBP-based AD. Then, the DTBP-based AD, in a single-filter platform, was washed twice with 5 mL of PBS using a syringe to remove debris and residual contaminants. Next, DNA was extracted using 100 μL of elution buffer consisting of 10 mM sodium bicarbonate (pH < 10.6) by breaking the crosslinking with the DTBP and amine group of APDMS within a few minutes.

### Biological samples

Human hepatocellular carcinoma (HepG2), prostatic adenocarcinoma (LNCaP), breast cancer (MCF-7), and myelogenous leukemia (K-562) cells were purchased from the Korean Cell Line Bank (Seoul, Korea). Colorectal carcinoma (HCT-116; ATCC CCL-247) cells were cultured in Dulbecco's modified Eagle medium (Life Technologies, Carlsbad, CA, USA), and human HepG2, LNCaP, MCF-7, and K-562 cells were cultured in RPMI 1640 medium (Gibco, Thermo Fisher Scientific Ltd., Waltham, MA, USA). Cells were maintained in a 75 cm^2^ flask containing medium supplemented with 10% fetal bovine serum (Gibco) and 1% penicillin/streptomycin (Gibco) at 37 °C in a 5% CO_2_ atmosphere. When cells reached approximately 80% confluence, they were detached using trypsin/EDTA (Gibco) and harvested after centrifugation at 1200 rpm for 3 min at RT. Cells were counted and lysed in a 200 μL lysis buffer (100 mM Tris–HCl [pH 8.0], 10 mM ethylenediaminetetraacetic acid, 1% sodium dodecyl sulfate, and 10% Triton X-100). The lysates were centrifuged at 13,000 rpm for 3 min at 4 °C, and then the supernatant was transferred to a new tube.

### DNA and protein analysis

The quantity and purity of the DNA were measured using a previously described procedure^18^. Genomic DNA was analyzed by PCR using in-house primers for *β-actin* and *TGF-β* (Table S3). PCR analysis was performed using a previously described procedure^18^. The quantity of separated proteins from cancer cells using the DTBP-based AD syringe filter platform was assessed using the Bradford assay to verify protein separation. The separated proteins were evaluated by Western blot for β-actin (Abcam, Cambridge, UK) and TGF-β activated Kinase 1-binding Protein 1 (TAB1; Abcam), and silver staining. Western blotting and silver staining were measured using a previously described procedure^18^.

## Supplementary information

Supplementary Information 1.
