# Supplementary material for: Dimethyl 3,3′-dithiobispropionimidate-functionalized diatomaceous earth particles for efficient biomolecule separation
Source: Sci Rep. 2020 Sep 24;10:15592. doi: 10.1038/s41598-020-72913-8 (PMC7519118; doi:10.1038/s41598-020-72913-8)
Supplement: Supplementary file 1 — Supplementary Information 1. [file 41598_2020_72913_MOESM1_ESM.docx]

**Supplementary**

**Dimethyl 3,3′-Dithiobispropionimidate-Functionalized Diatomaceous Earth Particles for Efficient Biomolecule Separation**

Yoon Ok Jang, Geun Su Noh, Huifang Liu, Bonhan Koo, Zhen Qiao, and Yong Shin^*^

Department of Convergence Medicine, Asan Medical Institute of Convergence Science and Technology (AMIST), University of Ulsan College of Medicine Biomedical Engineering Research Center, Asan Institute of Life Sciences, Asan Medical Center, 05505, Seoul, Republic of Korea

*Correspondence: [shinyongno1@gmail.com](mailto:shinyongno1@gmail.com) (Y. Shin); Tel.: +82-2-3010-4193

**
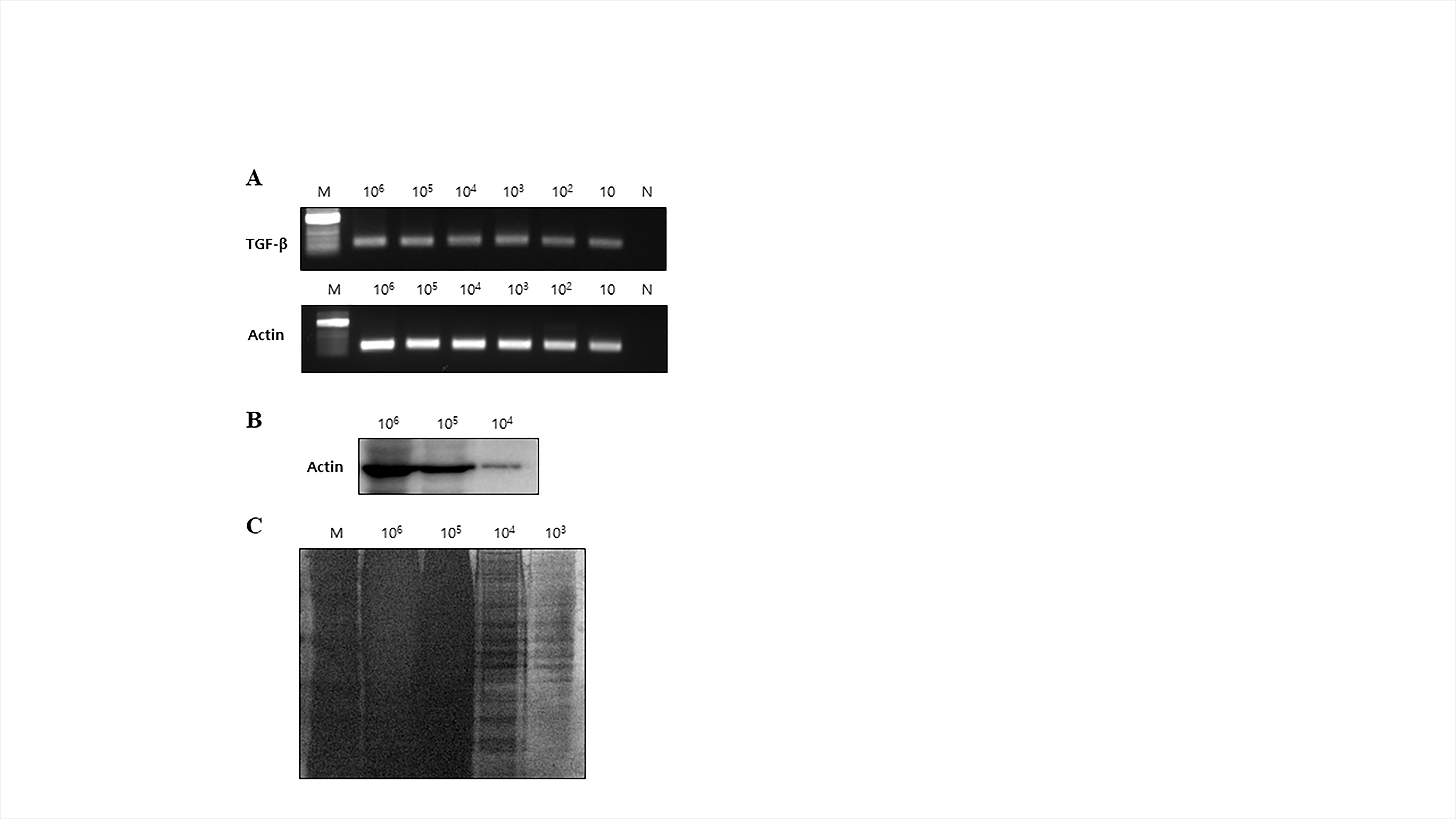
**

**Supplementary Figure 1.** Validation of the column-based system for DNA and proteins separation. A-C) Experiments comparing the performances of the column-based system with a series of LNCap cancer cell line, in the range of 10-10^6^ cells. The DNA extracted using the column-based method was analyzed by (A) PCR for *β-actin* and *TGF*-*β*. The proteins isolated by the column-based method were assessed by (B) Western blot for β-actin and (C) silver staining. (M: size marker, N: negative control).


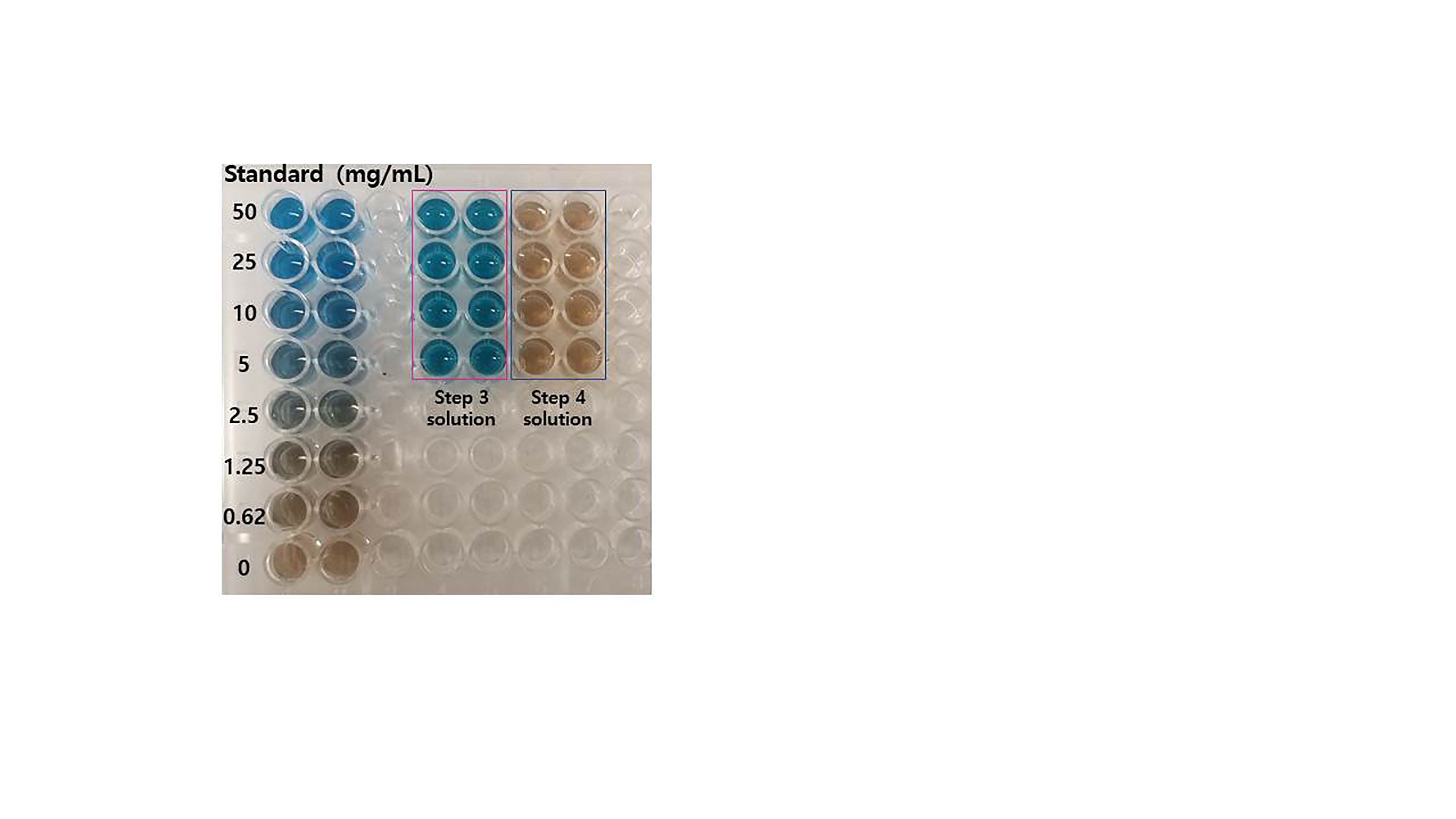


**Supplementary Figure 2.** The Concentration of proteins from proteins separation (step 3) and DNA separation (step 4) using Bradford protein assay.

**Table S1. The concentration of proteins and DNA using the DTBP-based AD syringe filter system**

| Cell | Cell concentration | Protein separation  (step 3) | DNA separation  (step 4) |
| --- | --- | --- | --- |
| HepG2 | 1X10^6^ | 26.6 µg/μL | 19.7 ng/μL |
|  | 1X10^5^ | 26.7 µg/μL | 17.4 ng/μL |
|  | 1X10^4^ | 24.6 µg/μL | 17.6 ng/μL |
| LNCap | 1X10^6^ | 29.7 µg/μL | 22.7 ng/μL |
|  | 1X10^5^ | 25.2 µg/μL | 17.5 ng/μL |
|  | 1X10^4^ | 24.0 µg/μL | 18.2 ng/μL |
| HCT-116 | 1X10^6^ | 26.5 µg/μL | 20.8 ng/μL |

**Table S2. The comparison of performance of the assays**

| Cell | Cell concentration | DTBP-based AD syringe filter | | Column based | |
| --- | --- | --- | --- | --- | --- |
|  |  | Protein  (µg/μL) | DNA (ng/μL) | Protein  (µg/μL) | DNA (ng/μL) |
| LNCap | 1X10^3^ | 14 | 15.8 | 0.49 | 5.5 |
|  | 1X10^2^ | 12.4 | 17.2 | 0.51 | 1 |
|  | 1X10 | 170 | 20.7 | 0.47 | 1.2 |

**Table S3. Primer sequences for PCR**

| Gene | Forward/reverse | Primer sequence |
| --- | --- | --- |
| *β-actin* | Forward | \| 5′-ATTGCCGACAGGATGCA-3′ \| \| --- \| |
|  | Reverse | \| 5′-CATACTCCTGCTTGCTGATCC-3′ \| \| --- \| |
| *TGF*-*β* | Forward | \| 5'-TTTTGATGTCACCGGAGTTG-3' \| \| --- \| |
|  | Reverse | \| 5'-AACCCGTTGATGTCCACTTG-3' \| \| --- \| |
